# From speciation to introgressive hybridization: the phylogeographic structure of an island subspecies of termite, *Reticulitermes lucifugus corsicus*

**DOI:** 10.1186/1471-2148-8-38

**Published:** 2008-02-04

**Authors:** Thomas Lefebvre, Nicolas Châline, Denis Limousin, Simon Dupont, Anne-Geneviève Bagnères

**Affiliations:** 1 I.R.B.I., CNRS UMR 6035, Université François Rabelais de Tours, Faculté des Sciences et Techniques, Parc de Grandmont, 37200 Tours, France; 2 L.E.S.T., IRD UMR 137, Université Paris XII – Val de Marne, 94010 Créteil cedex, France; 3 L.E.E.C., CNRS UMR 7153, Université Paris 13, 99 avenue J.B. Clément, 93430 Villetaneuse, France

## Abstract

**Background:**

Although much research has been carried out into European *Reticulitermes *taxonomy in recent years, there is still much discussion about phylogenetic relationships. This study investigated the evolution from intra- to interspecific phylogeny in the island subspecies *Reticulitermes lucifugus corsicus *and threw new light on this phenomenon. An integrative approach based on microsatellites and mitochondrial and nuclear DNA sequences was used to analyze samples taken from a wide area around the Tyrrhenian sea and showed how the subspecies evolved from its origins to its most recent form on continental coasts.

**Results:**

According to mitochondrial phylogeny and molecular clock calculations, island and continental taxa diverged significantly by vicariance in the Pleistocene glacial period. However, more recently, numerous migrations, certainly human-mediated, affected the structure of the populations. This study provided evidence of direct hybridization and multiple introgressions which occurred in several hybrid areas. Analysis using STRUCTURE based on microsatellite data identified a population in Provence (France) which differed considerably (Fst = 0.477) from populations on the island of Corsica and in Tuscany in the Italian peninsula. This new population, principally distributed in urban areas, is highly heterogeneous especially within the ITS2 regions where homogenization by concerted evolution does not appear to have been completed.

**Conclusion:**

This study provides an unusual picture of genetic interaction between termite populations in the Tyrrhenian area and suggests that more attention should be paid to the role of introgression and human impact on the recent evolution of European termites.

## Background

The entomologist P. Rossi [1] described the first subterranean termite species in Tuscany in 1792. The *Reticulitermes lucifugus *species was first called *Termes lucifugum *and for a long time was the only recognized species in Europe. Since then, extensive studies of the *Reticulitermes *genus in this region have shown the existence of six sibling species and a Tyrrhenian subspecies [2]. The precise classification of taxonomic groups is still of current interest and a matter of priority firstly for economic reasons and the need to define urban pests and secondly, of more fundamental importance, to reveal more information about the phylogenetic and biogeographical relationships in the genus [3-10].

*Reticulitermes lucifugus corsicus*, a subspecies of a European subterranean termite [2], is a species that may help to explain current issues. Although it was established that these termites lived exclusively on islands, recent studies have found the species on the mainland, in Tuscany (Uva *et al*. found the species in two urban areas [8] and Marini and Montovani found it in one urban area [4]) and in Provence [this study]. The hitherto unsuspected presence of the species on the continent raises certain questions about the characteristics specific to the Corsican termite, in particular its actual taxonomic status and the way in which it migrated to the continent. Its distribution across Tuscany and interaction with Italian termites need to be studied.

*R. lucifugus corsicus *has still not been clearly defined and is known from subsidiary descriptions in general publications [2,11,12]. As it is closely related to the Italian subspecies and as there is no evidence of reproductive isolation between the two taxa, *R. lucifugus corsicus *has been classified as an endemic subspecies of the islands of Corsica and Sardinia. Biometric data clearly puts the Corsican termites closer to continental *R. lucifugus *and only quantitative differences can be noted from chemical data – defensive secretions of the soldiers [13] and cuticular hydrocarbon proportions [2,14]. Only the yellow color of the tibia of reproductives is a significant morphological indicator of their Corsican origin, distinguishing them from *R. lucifugus *[2]. The best identification method remains the comparison of DNA sequences [3,4,8], as mitochondrial or nuclear haplotypes clearly distinguish the two taxa.

These characteristic differences probably developed after isolation on the islands and confirm that the taxon is endemic. However, it is difficult to date accurately at what stage *R. lucifugus corsicus *diverged from the Italian *R. lucifugus*. The geological history of the area is very complex [15-17]. The main hypothesis put forward by Plateaux and Clément [18], later supported by Uva *et al*. [8] and Luchetti *et al*. [5] is that the speciation event dates from the last glaciation period with a classic scenario of vicariance and post-glacial colonization. Any questions about the biogeographical history of the island subspecies can be answered by studying the development of termite colonies on the continent. This sympatric existence, in which hybridization is possible, can be considered as a real window on evolutionary processes [19,20] in the *Reticulitermes *genus. This area contains populations of termites that could possibly shed light on the past and recent evolution of the species complex, from the first speciation events to possible hybridization. More than two centuries after Rossi's discovery, Tuscany could once more be a region that may provide useful information for the study of European termites.

This phylogeographic study in the Tyrrhenian area reveals how the island *Reticulitermes lucifugus corsicus *termite evolved, from its origins to its recent appearance in continental coastal areas. An integrative approach was used to clarify the phylogenetic relationship with the Italian *R. lucifugus *termite and to detect any hybrid forms, using various genetic markers [21], such as mitochondrial and nuclear DNA sequences, and several microsatellite regions. These markers have different mutation rates and can, therefore, provide information about evolutionary events on different time scales.

## Results

### Phylogeny based on mitochondrial genes

**Figure 1:**
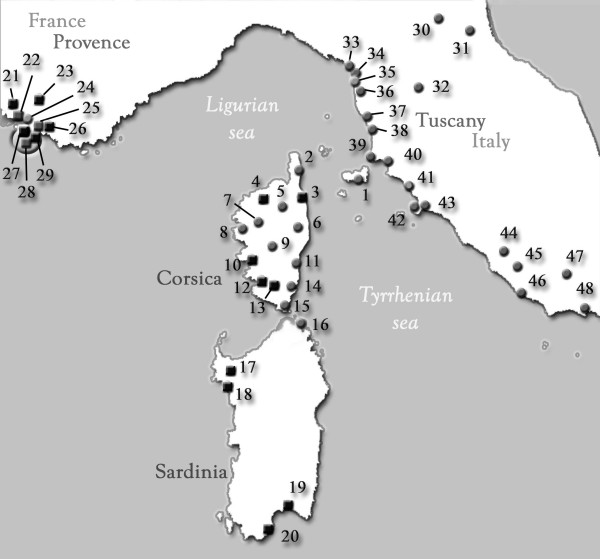
**Map showing the location of samples in the Tyrrhenian region**. Square points correspond to urban sites and round points to natural sites. Numbers refer to Table 1.

**Figure 2:**
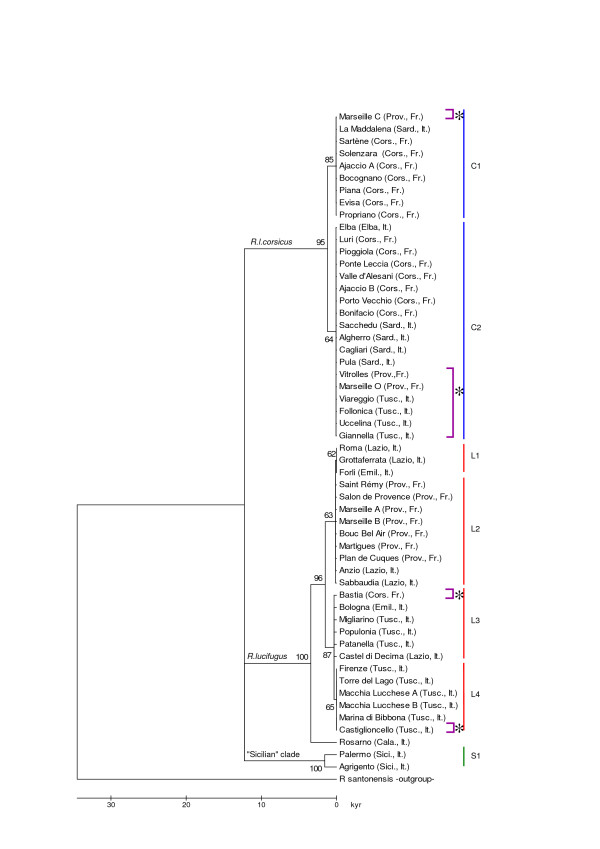
**Phylogenetic tree based on the Neighbor-Joining method, linearized to show the time of divergence**. The scale bar represents the time scale (Kyr) according to the geological calibration proposed by Luchetti *et al*. (2004). Bootstrap values are given above branches. Clades were scored on the right. C = *R. l. corsicus*. L = *R. lucifugus*. S = "sicilian" *R. lucifugus*. Purple brackets followed by an asterisk mark colonies found in unexpected clades.

The COII sequences of 60 samples (except Vitrolles B; see Table 1 and Figure 1) were aligned and no length polymorphism was detected. Forty-four of the 677 base pairs (bp) were variable and 35 were parsimony-informative. Phylogenetic analyses performed on 54 samples either with maximum parsimony (MP) (not shown; CI = 0.928, RI = 0.988) or with Neighbor-Joining (NJ) methods (Figure 2) gave the same topology. Two major clades were supported by high bootstrap values of 95% and 100% respectively. They correspond to the *lucifugus *(L) and *corsicus *(C) taxa matching sequences already in Genbank [4,5] and used for this study (Table 1). Twenty-one private substitutions distinguished the first two groups (p-distance = 0.0347, Table 2). The Sicilian sequences (Palermo and Agrigento) form a third clade (S) with the same degree of divergence from the L and C clades (Table 2). Twenty-two nucleotides separated the consensus sequence of the L clade, 20 compared to Rosarno (Calabre, South Italy) and 26 compared to Viareggio (Tuscany, Italy).

**Table 1:** Observed haplotypes, Genbank references and collection locations of samples used in this study.

Map codes	Localities	Haplotype		Genbank accession number	
			
		*COII*	*ITS2*	*COII*	*ITS2*
1	Elba	C2	C	EF591525	EF591552
2	Luri	C2	C	EF591533	EF591553
3	Bastia	L1	L	EF591507	EF591540
4	Pioggiola	C2	-	EF591534	-
5	Ponte Leccia	C2	-	EF591524	-
6	Valle d'Alesani	C2	L	EF591535	EF591541
7	Evisa	C1	-	EF591519	-
8	Piana	C1	-	EF591517	-
9	Bocognano	C1	-	EF591518	-
10	Ajaccio				
	- A	C1	C	EF591516	EF591554
	- B	C2	C	EF591523	EF591555
11	Solenzara	C1	C	EF591515	EF591556
12	Propriano	C1	-	EF591520	-
13	Sartène	C1	C	EF591521	EF591557
14	Porto-Vecchio	C2	-	EF591536	-
15	Bonifacio	C2	-	EF591538	-
16	La Maddalena	C1	C	EF591537	EF591558
17	Sacchedu	C2	-	AF291730	-
18	Algherro	C2	-	AF291729	-
19	Cagliari	C2	C	EF591539	EF591559
20	Pula	C2	-	AY267861	-
21	St Rémy	L2	L	EF591498	EF591542
22	Salon de Provence	L2	L	EF591501	EF591543
23	Plan de Cuques	L2	H*	EF591500	EF591563
24	Martigues	L2	H*	EF591497	EF591565
25	Bouc Bel Air	L2	L	EF591502	EF591544
26	Vitrolles				
	- A	C2	H	EF591499	EF591567
	- B	-	H**	-	EF591568
27	Marseille				
	- A	L2	L	EF591494	EF591545
	- B	L2	H*	EF591495	EF591564
28	- C	C1	C	EF591496	EF591560
29	- O	C2	H	EF591493	EF591566
30	Bologna	L3	-	AF291723	-
31	Forli	L1	-	AF291725	-
32	Firenze	L4	-	AF291726	-
33	Torre del Lago	L4	L	EF591514	EF591546
34	Macchia Lucchese				
	- A	L4	-	EF591511	-
	- B	L4	-	EF591512	-
35	Viareggio	C2	C	EF591522	EF591561
36	Migliarino	L3	-	EF591509	-
37	Castiglioncello	L4	C	EF591513	EF591562
38	Marina di Bibbona	L4	-	EF591510	-
39	Populonia	L3	-	EF591508	-
40	Follonica	C2	L	EF591503	EF591547
41	Uccelina				
	- genbank	C2	-	AF291727	-
	- 0	C2	-	EF591526	-
	- 1	C2	-	EF591527	-
	- 2	C2	-	EF591528	-
	- 3	C2	L	EF591529	EF591548
	- 4	C2	-	EF591530	-
	- 5	C2	-	EF591531	-
42	Giannella	C2	L	EF591532	EF591549
43	Patanella	L3	-	AF525341	-
44	Roma	L1	L	AF291729	EF591550
45	Grottaferrata	L2	-	EF591504	-
46	Anzio	L2	-	EF591505	-
47	Sabbaudia	L2	-	EF591506	-
48	Castel di Decima	L3	L	AF291724	EF591551
49	Rosarno	L5	-	AY267863	-
50	Palermo	S1	-	AY267857	-
51	Agrigiento	S1	-	AY267864	-

The haplotype code refers to phylogeny or sequencing alignments obtained according to the different nuclear or mitochondrial markers. C = *R. l. corsicus*. L = *R. lucifugus*. S = "sicilian" *R. lucifugus*.

**Table 2:** Genetic distance and diversity of clades defined by the phylogenetic analyses on COII sequences.

	Mean distance between clades			Mean diversity
	L	C	S	
*lucifugus*	-	-	-	0.00315
*corsicus*	0.0347	-	-	0.00445
"*sicilian*"	0.0378	0.0298	-	0.00175

These results refer to figure 2.

Several haplotypes were identified in the two main clades in this study, supported by bootstraps higher than 60%. Therefore, with only two subgroups (C1 and C2) found in many different localities, clade C had lower genetic variability than clade L (sequence diversity within clade C = 0.001750 vs. clade L = 0.003148, Table 2). The haplotype C2 was the most common. It was found on all the Tyrrhenian islands (including Elba) as well as in several samples from Provence and Tuscany which, a priori, were geographically non-concordant (Vitrolles, Marseille-O, Uccelina, Follonica, Giannella). Similarly, the other Corsican haplotype (C1) found in the Marseille-C sample could possibly be a continental introduction or introgression. Within clade L, termite colonies from Bastia were also included in an unexpected group from Tuscany (L3) and all the French colonies were linked with colonies from Lazio, South Rome (L2 and some samples of L1).

### Nuclear sequence analysis

**Figure 3:**
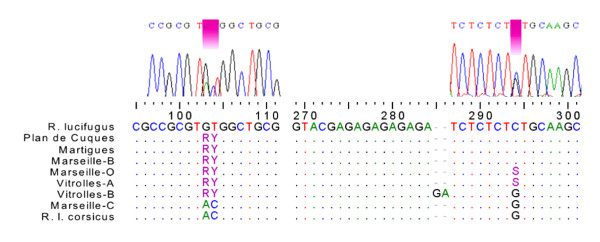
**Chromatograms and ITS2 sequences of Provencal hybrid colonies**. The three diagnostic nucleotides are shown to distinguish between the taxa. The letters 'R', 'Y' and 'S' indicate the presence of mixed forms, as found on upper chromatograms.

The nuclear sequences (ITS2), which generally evolve more slowly than the organelle genome [22,23], were far less variable with only 3 nucleotides of the 372 that differentiate the two taxa (Figure 3). Colonies from Follonica, Gianella, Uccelina (Tuscany, Italy) and Valle d'Alesani (Corsica, France) were included in the *'lucifugus' *clade, which was contrary to their mitochondrial data. This was also the case for the Castiglioncello colony (Tuscany, Italy), which had a 'Corsican' nuclear haplotype. This lack of concordance between nuclear and cytoplasmic genotypes could be an example of the asymmetrical introgression already described by Avise et al [24].

The nuclear data shows evidence of hybridization. Both taxa (Marseille-A: *R. lucifugus *and Marseille-C: *R. l. corsicus*) were found in Marseille (Provence, France) as well as a colony with a mix of both haplotypes for the same individual (Marseille-O, Figure 3). The sequence electropherogram (Figure 3) clearly revealed an overlap of the two peaks corresponding to the private substitution of each taxon at each variable site. The absence of noise in the areas round these peaks excluded the possibility of artefacts [25]. This was not an isolated case in Provence (Vitrolles-A). Other colonies in Provence had even more complex forms, either with a crossing-over type mutation on the third diagnostic locus (Marseille-B, Martigues, Plan de Cuques, Venelles) or the insertion of a microsatellite repeat (Vitrolles-B) (Figure 3).

### Microsatellite analysis

**Figure 4:**
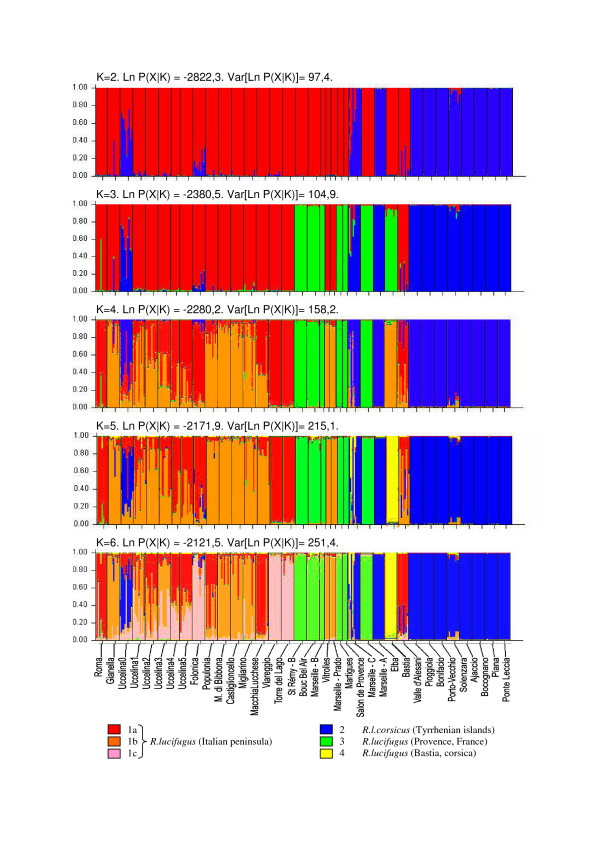
**Estimated population structure using microsatellite genotyping**. Analyses were performed using the admixture model provided by STRUCTURE software (Pritchard *et al*. 2000). A thin vertical line represents individuals of a colony. Each is probabilistically assigned to *K *populations (colored segments). Bar plotting was performed for *K *from 2 to 6. The change in the estimated likelihood values lnP(X|K) is given for each step.

Five microsatellite loci were analyzed. Heterozygosis was often low (Mean Ho = 0.16) with a mean of 3.83 alleles per locus (Additional file 1). These microsatellite regions were identified in phylogenetically distant *Reticulitermes *species [26] and seemed to have lower diversity in the Transtyrrenian specific complex. However, analysis of this data using structure [27] revealed significant differences between the populations. It can be difficult to determine the population structure of termites in a sample [28,29] and so the number of populations was estimated using our own biological appreciation [30]. Using the probable estimated values ln P (X|K) and the graphic results (Figure 4), the data could be structured in 3 to 4 populations. Histograms were usually incoherent from K = 7. For K = 2, STRUCTURE application distinguished both taxa. The Corsican termites from Valle d'Alesani could be classified according to their nuclear sequences (ITS2) among the *R. lucifugus*. Uccelina-0 (Q_2_.moy = 0.524), and Follonica (Q_2_.moy = 0,159) in Tuscany may both originate from an admixture between subspecies. The second run (K = 3) showed a new population nested in the *R. lucifugus *population in Provence, France. The Bastia colony (Corsica) formed a separate cluster for K = 4 and particularly for K = 5. The Marseille-C colony was partly assigned to this Bastia group (Q_4_.moy = 0,237). Finally, by over-estimating K, the program attributed colonies to populations. This could indicate genetic variability within the *R. lucifugus *peninsular group. However, this illustrated the homogeneity in the populations in the island samples (from Valle d'Alesani to Ponte Leccia, and Elba), colonies that had apparently been introduced (Bastia) and colonies in Provence (from St Rémy to Salon de Provence, and Marseille A).

**Figure 5:**
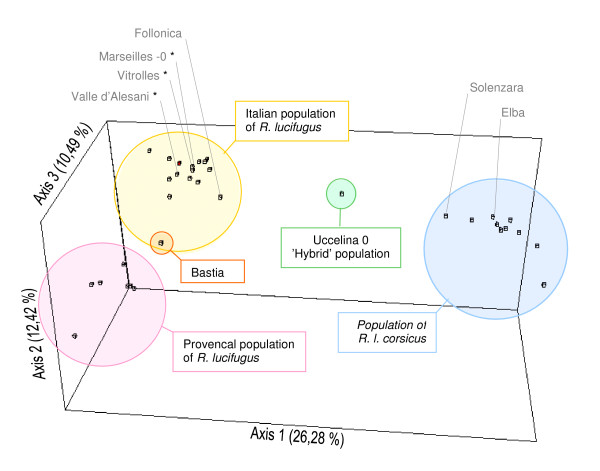
**Factorial correspondence analysis from microsatellite data**. The point clouds corresponding to genotyped colonies were assigned to different populations according to their assembling in space. Some colonies have been marked; those whose presence is contradictory to their geographical position were marked with an asterisk.

The results obtained using factorial correspondence analysis (FCA) also showed a similar distribution into 4 populations (Figure 5). Axis 1 (26.28%) distinguished *R. lucifugus *from *R. l. corsicus*. A hybrid colony (Uccelina) also appeared between the two groups. Axes 2 (12.42%) and 3 (10.49%) separated *R. lucifugus *colonies into two populations: one from Italy and one from Provence. Close to these clusters, the Bastia colony formed a separate population which was much more similar to the population in Italy. Finally, the *lucifugus *Italian group included colonies originating from unexpected geographical regions such as Valle d'Alesani (Corsica), Vitrolles-A and Marseille-O (Provence). Based on these results, the genetic differentiation between the three populations was calculated using F-statistics (Table 3). It appears that all the populations are highly distant to each other (Fst > 0.25). The population related to *R. l. corsicus *is the most differentiated population (mean Fst = 0,534), but, among *R. lucifugus *taxon, the Provencal population is also highly separated to the Italian population (Fst = 0.265).

**Table 3:** F-statistics (Fst) between different clusters defined by FCA for all populations from microsatellite data. These results refer to figure 5.

Fst between different clusters			
	1	2	3

*1-Italian*	-	-	-
*2-Provencal*	0.2649	-	-
*3-Corsican*	0.5442	0.5244	-

Fst for all population = 0.4777

## Discussion

### Past evolution: Origins of endemism

The mitochondrial phylogeny shows that the Tyrrhenian, Sicilian and mainland populations of *Reticulitermes *form three clearly distinct clades with a high level of divergence. The genetic distances between the island clades (C, S) and the mainland clade (L) are similar (mean distance ≈ 0.0350, Table 2). This suggests that the island populations (C, S) have a comparable evolutionary history. Today, very little is known about Sicilian populations of *Reticulitermes*. It would be interesting to investigate the level of endemism of these populations to see whether they could be considered as a subspecies, as has already been suggested [5].

The large number of mutations that have accumulated between the three clades shows a long-term separation without any interbreeding. The different hypotheses suggesting that all present-day European *Reticulitermes *taxa started to diverge from different southern Ice Age refugia [2,5,7,8,12,18] confirmed the postglacial colonization routes described by Taberlet *et al*. [31] and Hewitt [20]. More recently, Luchetti *et al*. suggested that Corsican and Sicilian populations differentiated later from the peninsular populations during the Last Glacial Maximum (LGM) period (18 kyr to 12 kyr) [32]. This time of divergence seems relatively recent in comparison to species-level differences observed in other taxa from these areas [20]. Some endemic taxa of the island of Corsica would have started to diverge from 0.01 to 0.08 Myr for mammals [33-35] and from 1.2 to 2.7 Myr for insects [36,37]. Many Corsican taxa may also have originated from the Messinian period (5 Myr) and earlier [38,39]. To justify the high rate of substitution seen in European *Reticulitermes *populations, Luchetti *et al*. argued that the social and geographical structure of populations could significantly increase the mtDNA substitution rate [32,40]. But the calibration of the molecular clock used by the authors and based on a geological event could also be questionable. Indeed, several marine regressions occurred in the early Pleistocene [15] which would have led to many junctions-disjunctions between the Tyrrhenian islands and the mainland. In our view, it is so possible that the present-day European *Reticulitermes *taxa have older origins than previously thought.

It is, however, difficult to determine whether dispersion or vicariance play a greater role in cladogenic events [41,42]. The geological history of the Transtyrrhenian area is highly complex [16,17] and does not support any particular theory. As mentioned above, many species of the Tyrrhenian area have different evolutionary and biogeographical histories. The possibility of an insular colonization from Tuscany after post-glacial expansion through the Italian peninsula has also been suggested [32]. Furthermore, the high level of genetic homogeneity (mitochondrial, nuclear and also microsatellite loci) observed in termite populations in Corsica could be the result of a founding effect initiated by a migratory and colonizing event. The endemic origin of *R. l. corsicus *is also a possibility, with ancestral origins from an isolated population in an Ice Age refugium. Thus, each taxon would have been separated from taxa from different refuges (in South Italy, in Sicilia, and somewhere in Corsica or in Sardania). This could explain why the three clades have almost the same number of substitutions (about 20 nucleotides) and consequently the same divergence time. As the Corsican mitotype is closer to the Calabran (19 nucleotides) than to the Tuscan (23 nucleotides), the populations in Corsica and Sardinian may have started to diverge before recolonization of Italy.

The expansion on the continent is confirmed by mitochondrial and microsatellite data. Colonization was slow and may have lasted several thousand years. At the mitotype level, this expansion through the Italian peninsula generated a South/North cline, already reported by Uva *et al*. [8]. The expansion rate was probably the same as for the Iberian *Reticulitermes*, as the divergence times are similar [6]. Moreover, subterranean termites disperse over short distances in natural conditions. For these reasons, Italian recolonization by *R. lucifugus *could correspond to a 'phalanx' expansion mode [43]. The microsatellite data (Figure 4) shows a clear allelic diversity, specific to continental populations (island-continental model [44]). However, with the exception of the Provencal area, the colonies form a homogeneous entity. This unusual structure could be attributed to a bottleneck event during the post-glacial colonization, particularly when crossing the Ligurian Alps. The mitochondrial haplotypes, similar to those in Rome could also suggest a more recent introduction, perhaps human-mediated, as this route was used by several civilizations.

### Recent evolution: Introductions, hybridization and introgression

In the more recent past, since 2000 BC, human civilization developed considerable marine traffic in the Tyrrhenian region. It is therefore highly probable that colonies of *Reticulitermes *were introduced into unexpected zones by human transport [2]. This kind of event has already been documented – *R. lucifugus *in Bordeaux (France) (personal communication Bagnères.), *R. grassei *in Devon (UK) ([45] and personal communication Bagnères), *R. santonensis *in Hamburg (Germany) and Santiago (Chile) [2], and *R. urbis *in France and Italy ([7] and Bagnères *et al*., unpublished results). Three colonies -Bastia, Viareggio and Marseille-C- appear to have been introduced. Of these, the colony in Bastia is of particular interest as it forms an entity, neither entirely Provencal (Figure 4, run 2) nor entirely Italian (Figure 4, runs 1 and 3). The two other introduced colonies may have crossed with autochtonous populations, as shown by traces of allochtonous genes in the microsatellite data.

More generally, migrations in the Tyrrhenian region have created different hybrid zones. They resemble the bimodal zones described by Jiggins and Mallet [46] and are characteristic of a quasi-absence of direct intermediate forms. Only the Uccelina-O colony has F1-type hybrids. Nevertheless, there are signs of varying degrees of hybridization in colonies such as the presence of unexpected genotypes.

Other authors [4,5,8] suggested that some hybrid zones were situated in natural areas in the Tuscany pine-woods. This study did in fact find some introgressive forms in Tuscan colonies – Parco del Uccelina, Reserve of Follonica, Castiglioncello and Gianella – with conflicting mitochondrial and nuclear haplotype data (Table 4). Although most of these Tuscan colonies have an allochtonous mitotype, introgression seems to act in both directions. Indeed, termites in Castiglioncello have Corsican nuclear sequences with an Italian mitotype. The fixation of allochtonous haplotypes can occur through backcrossing to the parental populations [21,47] or can be helped by biased sex-ratios already observed in *Reticulitermes lucifugus *[48,49].

**Table 4:** Description of genetic particularities observed in some localities for all markers considered.

	Type			
		
Localities	COII	ITS2	μsat.	Genetic particularities
				**Colony introduced without any detectable hybridization trace**
Bastia	**L**	**L**	**B**	Forms an population in its own
				**Colony introduced with hybridization traces**
Marseille C	**C**	**C**	**C**/B-I	Two merged colonies or only one hybrid colony
Viareggio	**C**	**C**	**I**	Autochthon microsatellite genotype
				**Autochthon colony with hybridization traces**
Valle d'Alesani	**C**	**C**	**I**/C	Mainly allochtonous microsatellite alleles
Solenzara	**C**	**C**	**C**/I	Some allochtonous microsatellite alleles
				**Asymmetrical introgression**
Castiglioncello	**L**	**C**	**I**	ITS2 regions in contradiction with the remaining genome
Follonica	**C**	**L**	**I**/C	Hybridization traces at the microsatellite loci level
Gianella	**C**	**L**	**I**	Mitochondrial *corsicus*-like genome linked to a nuclear *lucifugus*-like genome
Uccelina	**C**	**L**	**I**	
				**F1-type hybrid**
Uccelina 0	**C**	**-**	**C-I**	Heterozygosis at the microsatellite loci level
				**Provencal singular forms**
Martigues	**L**	**H**	**P**	
Marseille A, B	**L**	**H**	**P**	Cf. Figure 3
Plan de Cuques	**L**	**H**	**P**	Haplotype heterogeneity among the multiple copies of the ITS2 region
Vitrolles A	**C**	**H**	**L**	
Vitrolles B	**-**	**H**	**-**	

For ITS2 or COII haplotypes, C = *R. l. corsicus*, L = *R. lucifugus*, H = mixed forms. For microsatellite genotypes, B = Bastia, C = Corsica, I = Italy, P = Provence (letters correspond to populations defined by FCA).

On the opposite shore, the persistence of Corsican microsatellite alleles in the Follonica sample (Tuscany) and the presence of microsatellite alleles characteristic of Tuscany along the East coast of Corsica (Solenzara, Valle d'Alesani) could also indicate relatively recent hybridizations. In general, these traces of hybridization tend to diminish and even disappear by backcross with local populations.

In Southern France, the Provence region may be a specific new hybrid zone. Among the multiple copies of the ITS2 gene, some colonies (Figure 3) have a mix of haplotypes from the two origins, several forms of which come from DNA recombination. However, no signs of hybridization can be detected with microsatellite markers. Perhaps a higher number of microsatellite markers is required to improve detection of hybrid individuals [50]. The numerous variants in the sample may result from mechanisms such as unequal crossing-over and gene conversion during the concerted evolution, which may have homogenized the number of rDNA copies [51,52]. ITS sequence homogenization by concerted evolution has already been observed in a species of subterranean termites *Coptotermes gestroi *(Rhinotermitidae) by Jenkins *et al *[53]. The concerted evolution might currently be slow [54], and in the end slower than microsatellite loci homogenization by backcross or by genetic drift. This might explain why no apparent signs of hybridization are found in Provence. Nevertheless, current results do not exclude the possibility that these different forms come from an ancestral polymorphism, but the presence of both Corsican mitotype and Italian nuclear haplotypes in the Vitrolles A colony (Table 4) clearly supports the theory of a hybrid origin of ITS2 mixed forms.

The Provence region appears quite different from Tuscany and Corsica. Firstly, there is a distinct population entity, which is different from the Italian *R. lucifugus *and *R. l. corsicus *groups (Figures 4 and 5). Secondly, most of the colonies sampled were collected from urban areas (Figure 1). Thirdly, there are genetic particularities (mixed forms for ITS genes) not found anywhere else in the data (Figure 3). This raises the questions of whether these characteristics are correlated and whether the urban environment influences the termites' adaptation process and the evolution of hybrids.

## Conclusion

*Reticulitermes lucifugus corsicus *is one of the best-described subspecies of termite, defined by its distribution (Corsica, Sardinia, Elba and some continental localities), by various taxonomic criteria (genetic, morphological and chemical) and also by this complex phyletic evolution (see also the subspecies of *Reticulitermes speratus *[10]). Its divergence goes back to cladogenic events during the Ice Age. However, owing to many migratory events, the evolution of transtyrrhenian populations of termites has been more complex. The study carried out in the area revealed the existence of three very different populations (F_ST _= 0.477, Table 3): one population on the Italian peninsula (Tuscany), a second in the Tyrrhenian islands (Corsica) and a third in Provence. With the Sicilian phylum, they form strong cladistic units, constituting a complex of species within *R. lucifugus*. Although hybrids occur, it seems that systematic homogenization of the genomes occurs subsequently. With a few intermediate forms and even some introgressive forms, these taxa may maintain an identity signature, and may finally be on the way to speciation.

Nevertheless, evidence of introgression in hybrid zones could influence how *Reticulitermes *adapt. Allowing horizontal transfers of the genetic material, better adapted forms could appear in some small populations [21]. The colonies sampled in this study are small and patchily distributed in pinewood areas, especially in anthropized environments and could, therefore, fulfill the conditions necessary for such adaptation. From this point of view, it is important to consider the *Reticulitermes *as urban pests and study how they are adapting to cities – as for urban colonies in Provence. After thousands of years of speciation under paleogeographical constraints, human impact [2,5,55,56]-urbanism, maritime transport, and accidental infestations-seems to be the determining element in recent *Reticulitermes *evolution.

## Methods

### Sampling

Various *Reticulitermes *samples from the Tyrrhenian region were analyzed. Some samples had been collected recently in Corsica (France) and Tuscany (Italy), others came from old collections that had been kept in a laboratory (Uva 1999) or from ad hoc donations. Of the 61 collection sites detailed in Table 1, 42 were in natural areas near pine-woods and 19 were from infested urban sites. A transect was taken in Uccelina (Tuscany). A hundred specimens from each colony were stored in 96% alcohol for genetic analyses. Further information on the samples is given in Table 1 and the sites are shown on a map in Figure 1.

#### DNA extraction, amplification and sequencing

Total DNA was extracted from a single termite preserved in alcohol, using a modified version of the method described by Kocher *et al*. [57]. PCR amplification was performed using a Biometra 96 T1 thermal cycler or a Stratagene robocycler Gradiant 96. Different programs and amplification conditions were used depending on the types of primers used for sequencing or microsatellite analyses (Additional file 2).

Two types of sequencing commonly used to study the phylogeny of the *Reticulitermes *genus were used – mitochondrial sequences – cytochrome oxydase II gene (COII) [3,6,58]- and nuclear sequences – the ITS2 region (internal transcribed spacer) of the ribosomal DNA [6,45,58] (Additional file 2). Amplified DNA was purified using the Quiagen-PCR purification kit and sequenced in both directions. Each sequence generated by this study was deposited in GenBank database (the accession numbers are given in Table 1).

### Phylogenetic analysis

Multiple consensus sequences were aligned using the Clustal W algorithm [59] from the BIOEDIT 7.0.1 sequence editor [60] and corrected manually. Phylogenetic analyses of this data were performed using MEGA 3.0 [61]. DNA sequences were first analyzed using the neighbor-joining (NJ) method [62] on Kimura 2-parameter distances [63]. Maximum parsimony (MP) analyses were then conducted using a heuristic search method with 100 repeats and tree bisection-reconnection (TBR) with all characters unordered and unweighted. *R. santonensis *was chosen as the outgroup. Node support was estimated by searching on 1000 non parametric bootstrap replicates [64].

### Microsatellite genotyping

Ten termites from 36 Corsican and Tuscan colonies (including the Uccelina transect) were genotyped at six microsatellite loci previously isolated in the species *R. flavipes *[65] and *R. santonensis *[26](Additional file 1). PCR products were separated by electrophoresis on 6% polyacrylamide gels run on a LI-COR 4000 L sequencer. Alleles were scored using Gene Profiler 4.03 (Scanalytics, Inc.).

A factorial correspondence analysis (FCA) was then performed using genetix 4.05.2 software to classify the populations in different clusters [66]. genepop was used to calculate Fst values between the different clusters defined by the AFC [67]. STRUCTURE was then used for the genetic identification of clustered populations, allogenous introductions and potential admixed colonies [27]. This software uses a model-based clustering method to infer population structure using multiloci genotypes. Each individual in the colony was probabilistically assigned to K populations. Runs of various lengths were performed with different numbers of genetic clusters (K), testing all values of K from 2 to 7. Because the populations could potentially hybridize, the admixture model [27] provided by the software was used.

## Authors' contributions

TL did most of the sampling. TL, NC, DL and SD did the DNA sequencing and microsatellite genotyping. TL and NC analyzed the data and wrote the paper. AGB conceived and coordinated the study. All the authors read and approved the final manuscript.

## Supplementary Material

Additional file 1 **Analyzed microsatellite sequences characteristics and variability**. N = number of individuals, Na = number of polymorph alleles and Ho = mean heterozygosis rate observed for each locus in the *R. lucifugus *and *R. l. corsicus *colonies. Click here for file

Additional file 2 List of primers and PCR profiles used for amplification and sequencing. Click here for file
